# Molecular changes in the expression of human colonic nutrient transporters during the transition from normality to malignancy

**DOI:** 10.1038/sj.bjc.6600264

**Published:** 2002-04-22

**Authors:** D W Lambert, I S Wood, A Ellis, S P Shirazi-Beechey

**Affiliations:** Department of Veterinary Preclinical Sciences, University of Liverpool, Liverpool L69 7ZJ, UK; Department of Medicine, University of Liverpool, Liverpool L69 3GA, UK

**Keywords:** colon cancer, butyrate transport, glucose transport, gene expression

## Abstract

Healthy colonocytes derive 60–70% of their energy supply from short-chain fatty acids, particularly butyrate. Butyrate has profound effects on differentiation, proliferation and apoptosis of colonic epithelial cells by regulating expression of various genes associated with these processes. We have previously shown that butyrate is transported across the luminal membrane of the colonic epithelium via a monocarboxylate transporter, MCT1. In this paper, using immunohistochemistry and *in situ* hybridisation histochemistry, we have determined the profile of MCT1 protein and mRNA expression along the crypt to surface axis of healthy human colonic tissue. There is a gradient of MCT1 protein expression in the apical membrane of the cells along the crypt-surface axis rising to a peak in the surface epithelial cells. MCT1 mRNA is expressed along the crypt-surface axis and is most abundant in cells lining the crypt. Analysis of healthy colonic tissues and carcinomas using immunohistochemistry and Western blotting revealed a significant decline in the expression of MCT1 protein during transition from normality to malignancy. This was reflected in a corresponding reduction in MCT1 mRNA expression, as measured by Northern analysis. Carcinoma samples displaying reduced levels of MCT1 were found to express the high affinity glucose transporter, GLUT1, suggesting that there is a switch from butyrate to glucose as an energy source in colonic epithelia during transition to malignancy. The expression levels of MCT1 in association with GLUT1 could potentially be used as determinants of the malignant state of colonic tissue.

*British Journal of Cancer* (2002) **86**, 1262–1269. DOI: 10.1038/sj/bjc/6600264 www.bjcancer.com

© 2002 Cancer Research UK

## 

Colon cancer is a major cause of mortality in the industrialised nations. It is resistant to most cytotoxic drugs, and success in surgical resection depends largely on the absence of local and distant metastases. The majority of colorectal cancers develop from premalignant polyps, commonly referred to as adenomas (Muto *et al*, 1975). The transition from normality to malignancy through the adenoma-carcinoma sequence is characterised by an alteration in the expression of a number of genes associated with the maintenance of cellular homeostasis (Vogelstein *et al*, 1988). It is increasingly evident that specific dietary factors have the potential to influence the normal structure and function of the colon, and various dietary components have been proposed as being either beneficial or potentially harmful to colonic health (Lipkin *et al*, 1999). Epidemiological studies over the past 30 years have indicated that a diet high in fibre and resistant starch can protect against colon cancer (Burkitt, 1971; Kune *et al*, 1987).

Dietary fibre and resistant starch escaping hydrolysis in the small intestine are fermented by colonic microflora to the short-chain fatty acids (SCFA) acetate, propionate and butyrate (Macfarlane and Cummings, 1991). Butyrate has an important role in maintaining the health of the colonic mucosa (Treem *et al*, 1994; Bugaut and Bentejac, 1993). It is the preferred metabolic fuel of the healthy colonic epithelium, providing over 60% of the colonocytes' energy requirements (Cummings, 1984). In addition, it induces growth inhibition and terminal differentiation in a variety of human colon cancer cell lines (Kruh, 1982; Hague *et al*, 1995). These effects are correlated with changes in the expression of various genes associated with these processes (Archer *et al*, 1998; Siavoshian *et al*, 2000; Hague *et al*, 1997). *In vivo* studies have correlated butyrate levels with a decreased incidence of colon cancer (Clausen *et al*, 1991), and butyrate instilled into the colonic lumen was shown to reduce tumour development in a chemical model of colon carcinogenesis (Medina *et al*, 1988). Essential to these roles is the absorption of butyrate across the colonic luminal membrane into colonocytes. At the luminal pH of the human colon (pH 7), butyrate is present almost entirely in its anionic form (Phillips and Devroede, 1979). This charged species is unable to cross the colonocyte membrane by free diffusion, and therefore requires a specific transport protein (Ritzhaupt *et al*, 1998a). We have previously demonstrated that this carrier protein is the monocarboxylate transporter, MCT1 (Ritzhaupt *et al*, 1998b). MCT1 is a member of the monocarboxylate transporter family, of which nine isoforms have so far been identified (Halestrap and Price, 1999).

Under normal circumstances, glucose provides a small fraction of the energy requirement of the colonic epithelium (Roediger, 1982). Glucose is absorbed from the blood across the basolateral membrane of colonocytes via the low affinity glucose transporter GLUT2 (Pinches *et al*, 1993), a member of the widely expressed GLUT family of facilitative glucose transporters (Baldwin, 1993; Shirazi-Beechey, 1995). It has been reported, however, that colorectal cancers, in common with many other malignancies, have enhanced glucose utilisation and glycolytic metabolism (Eigenbrodt *et al*, 1985; Holm *et al*, 1995). In many cases this increase in glucose utilisation is often accompanied by expression of the high affinity glucose transporter, GLUT1 (Yamamoto *et al*, 1990). GLUT 1 expression is normally confined to erythrocytes and cells at the blood–brain interface, but is expressed by cells transformed with *ras* or *src* oncogenes in culture (Flier *et al*, 1987) and by a number of malignant cell types (Brown and Wahl, 1993; Nagase *et al*, 1995; Mellanen *et al*, 1994).

In this study we have used immunohistochemistry and *in situ* hybridisation histochemistry to elucidate the pattern of expression of the colonic butyrate transporter, MCT1, along the crypt–surface axis. We report that the level of MCT1 mRNA and protein is significantly reduced in colonic adenomas and carcinomas, with the most dramatic decline observed in poorly differentiated carcinomas. We propose that a decline in the expression of MCT1 results in a reduction in the intracellular concentration of butyrate required to regulate cellular homeostasis, and to be used as the primary source of energy in the colonic epithelium. In the majority of carcinomas studied, the suppression of MCT1 expression is accompanied by the expression of the high affinity glucose transporter, GLUT1 and a down-regulation of the low affinity glucose transporter, GLUT2. We suggest that this rearrangement in gene expression may provide the tumour cells with a growth advantage. The levels of expression of MCT1 in association with GLUT1 and GLUT2 could provide markers for improved diagnosis and tumour classification. Furthermore, a better understanding of the underlying mechanisms involved in the changes in nutrient transporter gene expression could lead to the identification of potential therapeutic targets.

## MATERIALS AND METHODS

### Tissue samples

#### Tissue sections

Paraffin embedded archival colonic tissue sections from both male and female patients aged 54–84 years were provided by the Royal Liverpool University Hospital Tissue Bank. Sections were also prepared from biopsies obtained from male and female patients (aged 53–82) undergoing colonoscopy. Together they consisted of sections from 25 healthy colon samples, 20 histologically graded adenomas, and 30 carcinomas.

#### Resections and biopsies

Segments of colonic tissues were obtained from 10 male and female patients (aged 53–82) undergoing surgery for colon carcinomas. The histologically normal boundaries were removed and designated as normal colonic tissue, whilst the remainder were identified as carcinoma. Biopsy samples were removed from various regions of the colon of 10 individuals, aged 35–82, undergoing routine examinations. The biopsies were shown to be histologically normal. After removal they were frozen rapidly in liquid nitrogen and subsequently stored at −80°C until use.

Approval was obtained from the Royal Liverpool and Broadgreen Hospitals Ethical Committee for the work presented in this paper. Informed written consent was secured from all individuals prior to removal of tissue.

### Antibodies

The antibody to MCT1 was raised in rabbits against a peptide (CQKDTEGGPKEEESPV) corresponding to the C-terminus region of human MCT1, based on the procedure described by Lachmann *et al* (1986). The GLUT1 antibody was a kind gift from S Baldwin (University of Leeds, UK). The GLUT2 and villin antibodies were from Biogenesis Ltd (UK) and The Binding Site (UK), respectively. Polyclonal antibodies to rat/human MCT2 and MCT4 were kindly provided by A Halestrap (University of Bristol, UK).

### Peroxidase immunohistochemical detection of MCT1 and GLUT1

Paraffin embedded sections (7 μm) were mounted on APES treated slides, dewaxed and rehydrated through graded ethanol solutions. The sections were microwaved in 1% ZnSO_4_ for 10 min and allowed to cool, before endogenous peroxidase activity was quenched by incubation in 0.3% hydrogen peroxide for 15 min. Non-specific antibody binding sites were blocked by incubation in 5% BSA for 1 h at room temperature. Primary antibodies were added at the following concentrations diluted in PBS : MCT1, 1 : 100- 1 : 200 and GLUT1, 1 : 100. Sections were incubated at 4°C overnight in a humid container, before being rinsed twice in PBS. Horseradish peroxidase-conjugated anti-rabbit secondary antibody (DAKO, UK) was added at a concentration of 1 : 200 in PBS and incubation carried out at 25°C for 1 h in a humid container. Sections were incubated in DAB solution (0.5 mg ml^−1^) for 10 min in the dark and counterstained with Haematoxylin Mayer (Raymond Lamb, UK). Coverslips were mounted in D.P.X (Raymond Lamb, UK) and the sections viewed and photographed.

### *In situ* hybridisation histochemistry

A plasmid (pGEM-T, Promega) containing a 545 bp cDNA fragment of MCT1 (Ritzhaupt *et al*, 1998b) was linearised to provide a template for subsequent *in vitro* transcription of both sense and antisense cRNAs. Complementary RNA was prepared from the linearised cDNA template using the DIG RNA labelling kit (Roche), according to the manufacturer's instructions. The labelled cRNA was precipitated overnight and its integrity confirmed by denaturing electrophoresis.

Tissue sections were de-waxed in 100% xylene before being hydrated in graded ethanol solutions to ddH_2_O. Hydrated sections were placed in 0.2 M HCl for 20 min, washed in 2×SSC twice for 3 min and rinsed in 0.05 M Tris/HCl, pH 7.4. Sections were incubated in proteinase K (1–15 μg ml^−1^, optimised for each sample) in an omnislide incubation chamber (Hybaid, UK) at 37°C for 1 h and rinsed twice in 0.2% glycine/PBS. Post-fixation was carried out in 4% paraformaldehyde/PBS for 4 min before the slides were rinsed in PBS and transferred to 20% acetic acid for 45 s to block endogenous alkaline phosphatase activity. Sections were rinsed in 0.1 M triethanolamine and immersed in freshly prepared 0.25% acetic anhydride, 0.1 M triethanolamine pH 8.0 for 10 min. At this stage, one slide was incubated in RNase (20 μg ml^−1^) at 37°C for 1 h, with the remaining slides maintained at room temperature in PBS. Following RNase pretreatment, all the sections were rinsed in ddH_2_O and incubated at 50°C in pre-hybridisation solution (50% formamide, 0.3 M NaCl, 20 mM Tris pH 8, 5 mM EDTA pH 8, 10 mM DTT, 1×Denhardt's solution, 1 mg ml^−1^ yeast tRNA) for 1 h in an Omnislide chamber. Hybridisation solution was added (50% formamide, 0.3 M NaCl, 20 mM Tris pH 8, 5 mM EDTA pH 8, 10 mM DTT, 1×Denhardt's solution, 1 mg ml^−1^ yeast tRNA, 100 mg ml^−1^ dextran sulphate, 200 ng ml^−1^ DIG-labelled cRNA) and incubation at 50°C continued overnight. Following hybridisation the sections were rinsed in 2×SSC, 1 mM DTT at room temperature and transferred to 2×SSC for 1 h. The slides were incubated at 50°C in wash buffer (0.3 M NaCl, 20 mM Tris/HCl pH 8, 1 mM EDTA, 1 mM DTT) overnight, washed in 2×SSC for 30 min at room temperature and transferred to 0.1×SSC for 30 min. Non-specific protein binding sites were blocked by incubation in 0.5% blocking buffer (Roche, UK) at room temperature for 30 min before incubation with anti-DIG antibody (Roche, 1 : 500 in blocking buffer) at room temperature for 2 h. Following incubation, the sections were incubated in detection buffer (100 mM Tris-HCl pH 9.5, 100 mM NaCl, 50 mM MgCl_2_, 0.18 mg ml^−1^ BCIP, 0.34 mg ml^−1^ NBT) and the reaction stopped when appropriate by immersion in ddH_2_O. The sections were counterstained with methyl green (0.5% (w v^−1^), 15 s), air-dried and coverslips mounted with Loctite UV adhesive.

### Preparation of post-nuclear membrane fractions from colonic biopsies

Human colonic tissue samples, 30–60 mg wet weight, from healthy (control) and carcinoma were homogenised using a polytron probe (6T microshaft, Ystral) with 500 μl of a buffer (100 mM mannitol, 2 mM HEPES/Tris, pH 7.1). The probe was washed with a further 250 μl of the same buffer, added to the suspension, and centrifuged at 500 **g** for 10 min (Sorvall RC5C). The supernatant was centrifuged at 30 000 **g** for 30 min and the resultant pellet was resuspended in a buffer containing 300 mM mannitol, 20 mM HEPES/Tris, pH 7.4, 0.02% NaN_3_, and made homogeneous with a Hamilton syringe. The protein concentration of membranes was determined by its ability to bind Coomassie blue according to the Bio-Rad assay technique (Tarpey *et al*, 1995).

### Western blotting

#### Assessment of MCT1 abundance

The abundance of MCT1 protein in post-nuclear membrane samples was determined by Western blotting as described previously (Dyer *et al*, 1997). The protein components of colonic membranes (10 μg per lane) were separated on 8% (w v^−1^) polyacrylamide gels containing 0.1% (w v^−1^) SDS and electrotransferred to nitrocellulose membrane (Trans-blot, Biorad, UK). Non-specific protein binding sites were blocked by immersion of the nitrocellulose in PBS-TM (PBS, 0.05% (v v^−1^) Tween 20, 2% (w v^−1^) non-fat dry milk) for 1 h at room temperature. The nitrocellulose membrane was incubated at room temperature for 1 h with the antibody to MCT1 diluted 1 : 5000 in PBS-TM. Horseradish peroxidase-conjugated anti-rabbit antibodies (Dako) were used at a dilution of 1 : 2000. The specific immunoreactive band (40 kDa) was blocked when the primary antibody was pre-incubated with the immunising peptide. Immunoblots were developed using the enhanced chemiluminescence (ECL) system (Amersham International, UK) and exposed to Biomax-ML film (Kodak). Band intensities were quantified by scanning densitometry (Phoretix 1D, Non-linear Dynamics).

#### Assessment of abundance of GLUT1, GLUT2 and villin

The nitrocellulose membrane used for Western blotting (see above) was stripped of anti-MCT1 antibodies by washing 3×10 min in an acidic buffer (137 mM NaCl, 20 mM glycine/HCl pH 2.5; Dyer *et al*, 1997). The membrane was rinsed in PBS containing 0.1 mM EDTA and 0.5% (v v^−1^) Triton X-100 (PBS-TE), and the samples blotted for GLUT1 using a 1 : 2000 dilution in PBS-TE. The stripping procedure was repeated after each blot and the samples blotted for GLUT2 (1 : 2000 in PBS-TE) and villin using a monoclonal antibody to villin (1 : 1000 dilution in PBS-TE). Horseradish peroxidase-conjugated anti-rabbit or anti-mouse secondary antibodies (DAKO) were used at a dilution of 1 : 2000 in PBS-TE. Specific immunoreactive bands for GLUT1 and GLUT2 (55 kDa) were blocked when the antibodies were pre-incubated with their respective immunising peptides. The immunoblots were developed as described above.

#### Assessment of MCT2 and MCT4 protein levels

The protein components of colonic post nuclear membranes (20 μg per lane) were separated on 8% (w v^−1^) polyacrylamide gels containing 0.1% (w v^−1^) SDS and electrotransferred to nitrocellulose membrane (Trans-blot, Biorad, UK). Immunoblotting was carried out as described above using antibodies to MCT2 and MCT4 (diluted 1 : 5000 and 1 : 1000 in PBS-TM, respectively).

### Northern blotting

Total RNA was isolated from healthy biopsies or of paired normal and carcinoma tissue samples (30–60 mg) using the RNeasy kit (Qiagen) according to the manufacturer's instructions. RNA samples, 10 μg per lane, were separated on 1% (w v^−1^) denaturing agarose gels and transferred to nylon membrane (Duralon, Stratagene, UK) in the presence of 1.5 M sodium chloride and 0.15 M sodium citrate (10 × SSC). The RNA was fixed to the membranes by UV light irradiation. RNA integrity and equality of loading/transfer were assessed by methylene blue staining of the membranes. The nylon membranes were pre-hybridised for 3 h at 42°C with ULTRAhyb hybridisation solution (Ambion, UK) followed by hybridisation for 16 h in the same buffer containing 1×10^6^ c.p.m. ml^−1^ of the appropriate cDNA probe labelled with [α-P^32^]dCTP using an Oligolabelling kit (Amersham Pharmacia Biotech). Subsequently, the membranes were washed three times at 55°C in a solution consisting of 0.1×SSC and 0.1% (w v^−1^) SDS. The washed membranes were exposed to Biomax-MS film (Kodak) for 24 h at −80°C and subjected to autoradiography. The relative amounts of mRNA were estimated by scanning densitometry of the autoradiograms (Phoretix ID quantifier, Non-linear Dynamics). The cDNA probes were as follows: MCT1, 545 bp RT–PCR product from human colon (Ritzhaupt *et al*, 1998b); GLUT1, 2.4 kb *Bam*HI fragment (cDNA kindly provided by S Baldwin, University of Leeds, UK); MCT2, 679 bp RT–PCR product corresponding to nucleotides 731–1409 (Lin *et al*, 1998); MCT4, 590 bp RT–PCR product corresponding to nucleotides 174–763 (Price *et al*, 1998); 18S rRNA cDNA was a gift from J Hesketh, University of Newcastle-upon-Tyne and a 1.4 kb restriction product was used as a probe.

### Statistics

Data are presented as means±s.e.m. of *n* samples. Comparisons are made using the Student's *t*-test.

## RESULTS

### Immunohistochemical detection of MCT1 in healthy colonic epithelium

**Figure 1:**
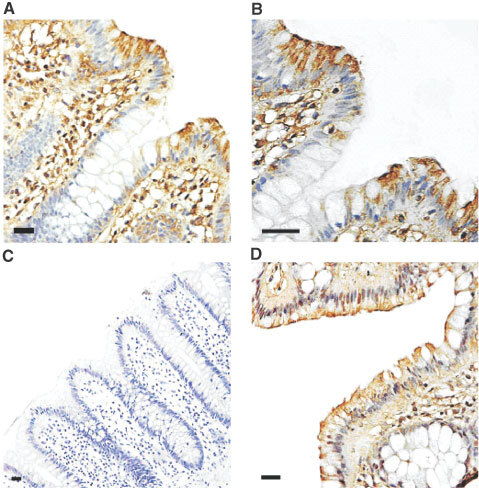
Immunohistochemical detection of MCT1 in healthy human colon. Immunohistochemistry for MCT1 and villin was carried out on healthy human colon biopsy sections using a peroxidase-conjugated secondary antibody as described in the Materials and Methods section. MCT1 staining is seen as a brown colouring contrasting with the blue/purple counterstained nuclei. (**A**) MCT1, ×200 magnification; (**B**) MCT1, ×400 magnification; (**C**) MCT1 antibody pre-incubated with immunising peptide, ×100 magnification; (**D**) immunodetection of villin, ×200 magnification. Bars represent 50 μm.

We have shown previously by immunoblotting that the butyrate transporter, MCT1, is enriched 30-fold in the luminal membranes isolated from healthy human colonic tissue compared to the cellular homogenate, indicating that the protein is predominantly located on the luminal membrane of human colonocytes (Ritzhaupt *et al*, 1998b). Here, using immunohistochemistry, we sought to confirm and extend this observation to investigate the pattern of MCT1 expression along the crypt-surface axis. Immunohistochemical detection of MCT1 using a horseradish peroxidase-conjugated secondary antibody confirmed that MCT1 is predominantly located on the luminal membrane of healthy colonocytes (Figure 1A

,B). The signal was blocked by pre-incubation of the antibody with the immunising peptide (Figure 1C). Immunodetection of villin, a marker of the colonocyte apical membrane, revealed a similar cellular localisation (Figure 1D). The level of expression of MCT1 was greatest on the luminal membrane of epithelial cells on the surface of the colonic mucosa, and declined rapidly with descent into the crypts (Figure 1A,B). At the base of the crypts only very low levels of MCT1 protein were detected. MCT1 is also present on the membranes of erythrocytes, contributing a degree of staining within the lamina propria.

### Detection of MCT1 transcript in colonic epithelium by *in situ* hybridisation histochemistry

**Figure 2:**
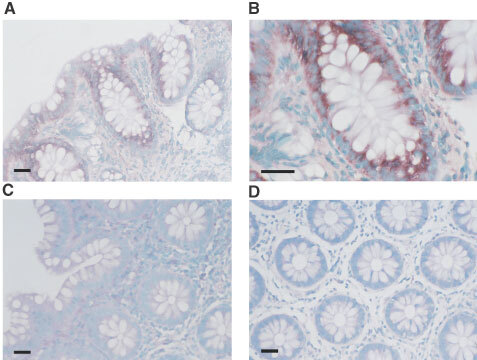
Detection of MCT1 mRNA in healthy human colonic biopsy sections by *in situ* hybridisation. *In situ* hybridisation was carried out on healthy human colon biopsy sections using DIG labelled cRNA probes as described in the Materials and Methods section. Sections were hybridised with a specific antisense probe for MCT1 and the corresponding sense probe to act as a control for non-specific binding. (**A**) MCT1 antisense probe (×200); (**B**) MCT1 antisense probe (×400); (**C**) sense probe (×200); (**D**) MCT1 antisense probe on RNase pre-treated sections (×200). Specific staining is seen as a dark purple colour, contrasting with the blue/green counterstained nuclei. Bars represent 50 μm.

We next sought to investigate the profile of expression of the MCT1 transcript along the crypt-surface axis. *In situ* hybridisation histochemistry, using a DIG-labelled antisense probe specific for MCT1, indicated that the MCT1 transcript is expressed along the length of the crypt-surface axis (Figure 2A

,B). Hybridisation of the antisense probe with sections pre-incubated with RNase A (Figure 2D) or untreated sections with the sense probe (Figure 2C) resulted in no detectable staining, indicating that the signal was specific. Levels of the MCT1 transcript are significantly higher than the protein levels detected at the base of the crypts, indicating that the expression of the MCT1 mRNA precedes the MCT1 protein during cellular differentiation along the crypt-surface axis.

### Immunohistochemical detection of MCT1 in healthy, premalignant and malignant colonic tissue

**Figure 3:**
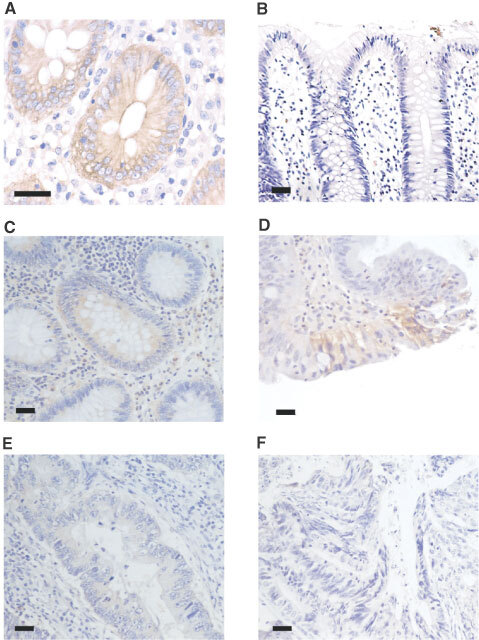
Immunohistochemical detection of MCT1 in normal, adenoma and carcinoma human colonic sections. Immunohistochemistry was carried out on normal, adenoma and carcinoma human colonic tissue using a horseradish peroxidase-conjugated secondary antibody as described in the Materials and Methods section. MCT1 staining is seen as a brown colouring contrasting with the blue/purple counterstained nuclei. (**A**) Healthy human colon (×400); (**B**) healthy human colon with MCT1 antibody pre-incubated with immunising peptide (×200); (**C**) tubular adenoma (×200); (**D**) tubular-villous adenoma (×200); (**E**) well differentiated carcinoma (×200); (**F**) poorly differentiated carcinoma (×200). Bars represent 50 μm. This data is summarised in Table 1.

Having established the profile of expression of the MCT1 transcript and protein in healthy colonic tissue, we next investigated the expression of MCT1 protein in sections of healthy colon, colonic adenomas and carcinomas. Sections were prepared from wax-embedded colonic tissues histologically graded as normal, tubular adenoma, tubulo-villous adenoma, and carcinoma of varying degrees of differentiation. A typical immunocytochemical analysis of healthy colonic tissue using peroxidase labelling is presented in Figure 3A

. High levels of MCT1 were expressed in the epithelium of all the healthy colon sections analysed. Of the adenoma samples analysed, tubular adenomas, which are generally regarded as having the lowest malignant potential, displayed the highest levels of MCT1 expression (Figure 3C), although in the majority of cases MCT1 abundance was still lower than in healthy colonic tissue. Tubulo-villous adenomas were found to express MCT1 at a much lower level than tubular adenomas, and the expression was limited to discrete foci (Figure 3D). MCT1 expression was significantly reduced in the majority of well-differentiated colonic carcinomas (Figure 3E), and was barely detectable in all the poorly differentiated colonic carcinomas analysed (Figure 3F). It should be noted that, for comparative purposes, the high antibody titre used (1 : 100, see Materials and Methods) resulted in cytoplasmic staining in the control samples expressing high levels of MCT1 (Figure 3A). The high antibody concentration was necessary to detect negligible levels of MCT1 in the carcinoma samples analysed. The data is summarised in Table 1

**Table 1:**
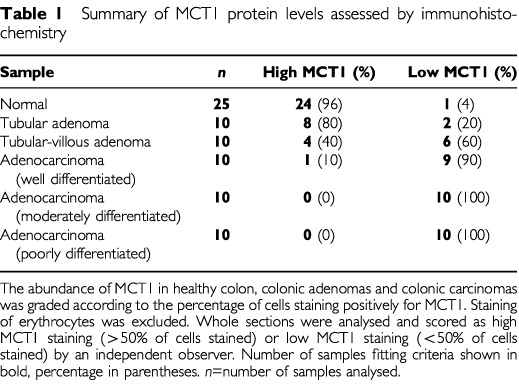
Summary of MCT1 protein levels assessed by immunohistochemistry

.

### Assessment of the levels of MCT1 protein and mRNA in healthy and colonic carcinomas by Western and Northern analyses

**Figure 4:**
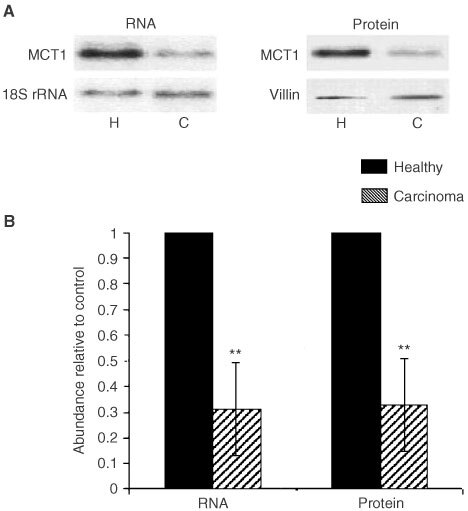
Assessment of MCT1 protein and mRNA levels in healthy colon and colonic carcinomas by Western and Northern analyses. Post-nuclear membranes and total RNA were isolated from normal and malignant colon, and analysed for MCT1, villin and 18S rRNA abundance as described in the Methods section. (**A**) Left panel: representative Northern blot showing abundance of MCT1 in healthy and malignant colon. Nylon membranes were stripped and reprobed for 18S rRNA to confirm equality of loading. Right panel: representative Western blot showing abundance of MCT1 in healthy and malignant colon. Nitrocellulose membranes were stripped and re-probed for villin to confirm equality of loading. (**B**) Combined densitometric analysis of Northern and Western blots displayed as a histogram. MCT1 abundance in carcinomas (shaded square) is displayed relative to healthy (solid square) where abundance in healthy samples=1. All values are normalised to the corresponding 18S rRNA or villin signals. Error bars represent±s.e.m. of 10 paired samples.

In order to assess quantitatively any potential changes in the levels of MCT1 mRNA and protein during progression from normality to malignancy, total RNA and post-nuclear membranes were isolated from healthy and malignant colonic tissues and analysed, by Northern and Western blotting respectively, for MCT1 expression. The levels of MCT1 mRNA in healthy and malignant colonic tissues were determined using Northern blotting. As shown in Figure 4A

(left panel), the levels of the 3.3 kb MCT1 transcript were significantly (68%±18, *P*=<0.001) reduced in the malignant tissue compared to the paired healthy tissue following normalisation to 18S rRNA controls. The expression of the 40 kDa MCT1 protein was found to be 67%±15 (*P*=<0.001) lower in carcinomas than in healthy colonic tissue (Figure 4A, right panel) when normalised to villin controls. The reduction in the level of MCT1 mRNA expression was comparable with the decline in MCT1 protein levels shown by Western blotting (Figure 4B).

### Determination of the levels of MCT2 and MCT4 protein and mRNA in colonic carcinoma

In healthy human colon, MCT2 is not expressed, whilst low levels of MCT4 mRNA have been detected (Price *et al*, 1998). Functional properties of MCT2 and MCT4 as elucidated by expression in *Xenopus* oocytes have indicated that these MCT isoforms are able to transport a range of monocarboxylates (Halestrap and Price, 1999).

**Figure 5:**
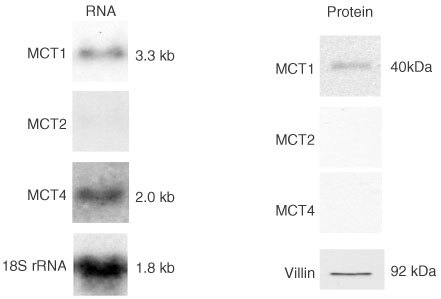
Assessment of the expression of MCT2 and MCT 4 in colon carcinoma measured by Western and Northern analyses. Post-nuclear membranes and total RNA were isolated from malignant colonic tissues, and analysed for MCT1, MCT2, MCT4, villin or 18S rRNA expression as described in the Methods section. Left panel: representative Northern blot showing abundance of MCT1 and MCT4 RNA in malignant colon. Nylon membranes were stripped and re-probed for 18S rRNA to confirm equality of loading. Right panel: representative Western blot showing the levels of MCT1 MCT2, and MCT4 proteins in malignant colon carcinoma tissues. Nitrocellulose membranes were stripped and re-probed for villin to confirm equality of protein loading. Note: specific immunoreactive bands were detected in control samples for MCT2 (in rat liver homogenate) and MCT4 (in rat heart tissue homogenate).

In colon carcinoma tissues MCT4 was expressed at the mRNA level, as assessed by Northern blotting (see Figure 5

). However, the MCT4 protein was not detected using either Western blotting (Figure 5) or immunocytochemistry. MCT2 was not expressed at either the mRNA or protein levels.

### Expression of GLUT isoforms in healthy and malignant colonic tissue

**Figure 6:**
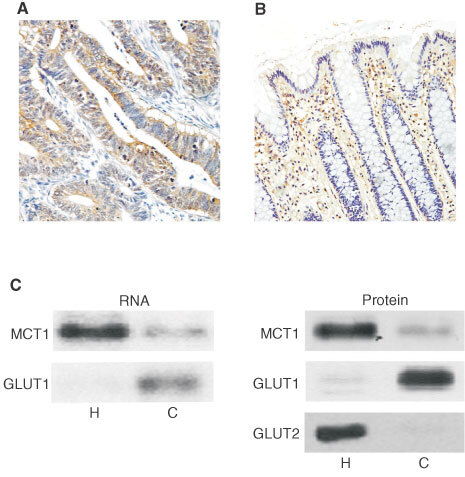
Abundance of GLUT1 in healthy and diseased colon. Immunohistochemistry was carried out on healthy and malignant human colonic tissue sections using a horseradish peroxidase conjugated secondary antibody as described under ‘Methods’. GLUT1 staining is seen as a brown colour contrasting with the blue/purple counterstained nuclei. (**A**) moderately differentiated carcinoma (×200). Bars represent 50 μm. Pre-incubation of antibody with immunising peptide resulted in absence of staining (data not shown). (**B**) Healthy colon (×200). Significant erythrocyte staining can be seen in the lamina propria. (**C**) Post-nuclear membranes and total RNA were extracted from healthy (H) and carcinoma (C) tissues and analysed for MCT1, GLUT1 and GLUT2 mRNA and protein abundance as described in ‘Methods’. Left panel: representative Northern blot indicating levels of MCT1 and GLUT1 mRNA (*n*=6). Right panel: Representative Western blot demonstrating levels of MCT1, GLUT1 and GLUT2 proteins (*n*=6).

Immunohistochemical analysis revealed that the majority (83.3%) of colon carcinoma samples displaying low levels of MCT1 expressed GLUT1 at significant levels (Figure 6A

). None of the healthy colonic tissue samples displayed detectable epithelial GLUT1 staining (Figure 6B). Staining of erythrocyte membranes is visible in healthy sections (Figure 6B), acting as an internal positive control. This data is summarised in Table 2

**Table 2:**
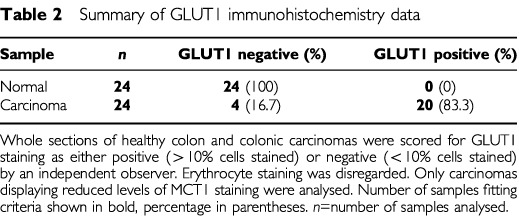
Summary of GLUT1 immunohistochemistry data

. This finding was reinforced by Western and Northern analyses, which indicated significant levels of both GLUT1 protein and mRNA in carcinoma samples displaying reduced MCT1 expression (Figure 6C). It is notable that the levels of GLUT2 protein, the low affinity glucose transporter normally expressed on the basolateral membrane of healthy colonocytes, were hardly detectable in all of the carcinomas expressing GLUT1 (Figure 6C, right panel; *n*=6).

## DISCUSSION

We have previously provided evidence that transport of butyrate into the absorptive cells lining the lumen of the colon is predominantly mediated by the monocarboxylate transporter, MCT1 (Ritzhaupt *et al*, 1998b), and the expression of colonic MCT1 is tightly regulated by its substrate, butyrate (Cuff *et al*, 2002). In this study we have sought to determine the *in situ* expression of MCT1 in the human colonic mucosa during the transition from normality to malignancy. In the healthy colonic epithelium, the MCT1 protein is primarily expressed on the apical membrane of colonocytes, with the abundance of the protein being greatest in surface epithelial cells and declining with descent into the crypts. The MCT1 transcript is expressed homogeneously along the crypt-surface axis, and is detectable in cells at the base of the crypts. A similar pattern of expression of mRNA and protein along the crypt-surface axis has been described for other intestinal nutrient transporters (Ramirez-Lorca *et al*, 1999; Gallardo *et al*, 2001), and may reflect the process of cellular differentiation from crypt to surface.

MCT1 protein abundance was significantly greater in healthy colonic tissue than in any tissue representing different stages of the adenoma–carcinoma sequence. Although lower levels of MCT1 were detected in the majority of tubular adenomas analysed than in healthy tissue, this level of expression was significantly higher than that seen in the majority of tubulo-villous adenomas analysed. Tubulo-villous adenomas are regarded as having a greater malignant potential than tubular adenomas (O'Brien *et al*, 1990), and this may be reflected in the reduction in MCT1 expression. The majority of carcinomas analysed exhibited very low levels of MCT1; indeed the protein was barely detectable in all the poorly differentiated carcinomas studied. It has been reported that MCT2 and MCT4 have the potential to transport a range of monocarboxylates (Halestrap and Price, 1999). It has been shown that in addition to MCT1, the high affinity pyruvate transporter MCT2 is expressed at the mRNA level in neoplastic haematopoietic lineage lines, Burkitt's lymphoma Raji, and in solid tumour cell lines such as SW480, A549 and G361 (Lin *et al*, 1998). The expression of MCT2 protein, however, in these cell lines has not been assessed (Lin *et al*, 1998), and therefore it is not evident if the mRNA is translated into the corresponding protein. In order to determine the potential presence of MCT2 and MCT4 in colon carcinoma, the expression of these isoforms, at both mRNA and protein levels, were assessed. Our results demonstrated that neither MCT2 nor MCT4 proteins are expressed; although MCT4 mRNA was detected. The antibodies to rat/human MCT2 and MCT4 however reacted with specific proteins in the control rat liver and heart homogenate samples, respectively. We conclude that MCT1 is the primary route for transport of butyrate in the human colon (Ritzhaupt *et al*, 1998a,b; Cuff *et al*, 2002), and that its expression declines dramatically during the transition from normality to malignancy.

The molecular mechanisms involved in the suppression of MCT1 expression during the transition from normality to malignancy are unknown. Work in our laboratory has shown that expression of MCT1 is regulated by butyrate via transcriptional and post-transcriptional mechanisms (Cuff *et al*, 2002). It has been reported that levels of butyrate in the lumen of the colon in patients with colorectal carcinomas is lower than in healthy individuals (Clausen *et al*, 1991). Whether the mechanism(s) involved in down-regulation of MCT1 expression is (are) due to the reduced substrate availability and/or the deregulation of intracellular events involved in MCT1 regulation remain to be determined.

A decline in the abundance of MCT1 in the membrane of colonic epithelial cells, and hence in butyrate uptake, would in turn reduce the availability of intracellular butyrate as a source of energy and as an important regulator of cellular homeostasis. Indeed, it has been reported that a reduction in the intracellular concentration of butyrate may lead to a deregulation of apoptosis and proliferation in colonic epithelial cells in culture (Singh *et al*, 1997). A down-regulation of MCT1 would also reduce the availability of butyrate to act as a cellular metabolite. It has been shown that colonic carcinomas switch to a more glucogenic phenotype and exhibit lower levels of fatty acid and ketone body utilisation (Holm *et al*, 1995). It is also documented that the colonic carcinoma cell line, Caco-2, has a high rate of glucose utilisation and glycogen content (Chantret *et al*, 1994). Many tumours, including colonic carcinomas, have been found to express the high affinity glucose transporter, GLUT1 (Yamamoto *et al*, 1990). Here, we show that 83.3% of carcinomas displaying significantly reduced levels of MCT1 express GLUT1. Notably, all of these tumours have negligible level of GLUT2, the low affinity glucose transporter expressed by healthy colonocytes. In cells that normally express GLUT2, such as hepatocytes and pancreatic islet β cells, oncogenic transformation induces *de novo* expression of GLUT1 (Thorens *et al*, 1988). Transformation is correlated with a decrease in the expression of GLUT2. GLUT1 has a significantly higher affinity for glucose (Km=1–2 mM; Wheeler, 1985) than GLUT2 (Km=15–40 mM; Craik and Elliott, 1979), suggesting that the expression of GLUT1 would allow cells to absorb glucose efficiently at low extracellular concentrations. In colonic tumours expressing low levels of MCT1, the induction of GLUT1 and the down-regulation of GLUT2 would enable the cells to take up and utilise glucose efficiently and ensure their growth and survival in the absence of their conventional energy source, butyrate.

In summary, our findings show that the colonic butyrate transporter, MCT1, is expressed predominantly on the apical membrane of surface colonic epithelial cells. Down-regulation of MCT1 expression occurs as an early event in the adenoma–carcinoma sequence and, in carcinomas, is associated with reduced abundance of the low affinity glucose transporter GLUT2 and the expression of the high affinity glucose transporter, GLUT1. These findings provide new potential markers of neoplastic change in the human colon. Furthermore, molecular insight into the mechanisms by which colonic tumours switch their energy source may allow rationally designed agents to block tumour growth and survival.
